# Brain Network Connectivity During Language Comprehension: Interacting Linguistic and Perceptual Subsystems

**DOI:** 10.1093/cercor/bhu283

**Published:** 2014-12-01

**Authors:** Elisabeth Fonteneau, Mirjana Bozic, William D. Marslen-Wilson

**Affiliations:** 1 Department of Psychology, University of Cambridge, Cambridge CB2 3EB, UK; 2 MRC Cognition and Brain Sciences Unit, Cambridge, UK

**Keywords:** gamma oscillations, MEG, morphology, speech comprehension

## Abstract

The dynamic neural processes underlying spoken language comprehension require the real-time integration of general perceptual and specialized linguistic information. We recorded combined electro- and magnetoencephalographic measurements of participants listening to spoken words varying in perceptual and linguistic complexity. Combinatorial linguistic complexity processing was consistently localized to left perisylvian cortices, whereas competition-based perceptual complexity triggered distributed activity over both hemispheres. Functional connectivity showed that linguistically complex words engaged a distributed network of oscillations in the gamma band (20–60 Hz), which only partially overlapped with the network supporting perceptual analysis. Both processes enhanced cross-talk between left temporal regions and bilateral pars orbitalis (BA47). The left-lateralized synchrony between temporal regions and pars opercularis (BA44) was specific to the linguistically complex words, suggesting a specific role of left frontotemporal cross-cortical interactions in morphosyntactic computations. Synchronizations in oscillatory dynamics reveal the transient coupling of functional networks that support specific computational processes in language comprehension.

## Introduction

To evaluate the incoming speech stream, the brain must analyze the sensory input and extract the relevant phonological, grammatical, and lexical information. This triggers neuronal activity that is distributed both anatomically and temporally across a large number of brain areas (Friederici 2002; Scott and Johnsrude 2003; Hickok and Poeppel 2007). A central question in cognitive neuroscience is to determine how these different computations and multiple distinct processing subsystems work together to produce a unified psycholinguistic percept (Pinker and Ullman 2002; Marslen-Wilson and Tyler 2007). Although neuroimaging research has revealed a rich constellation of regions involved in speech recognition, our understanding of the interactions between fast neuronal activities in the human cortex is still underdeveloped. Here, we capture the spatiotemporal patterns of neuronal activity evoked by specific linguistic computations, using noninvasive electro- and magnetoencephalographic (EMEG) recordings. We examine the functional oscillation synchrony of brain areas and reveal the cross-cortical communication that supports combinatorial linguistic computation in speech recognition. We argue that synchronization of neuronal oscillations may regulate cross-cortical communication, coordinating anatomically distributed neuronal activity that underpins speech comprehension (Fries 2009).

A particular hallmark of human language is its combinatorial property. The morpheme, the minimal combinatorial linguistic element, carries both semantic content (e.g., the stem *play*) and grammatical features (e.g., the affix *-ed*). Here, we focus on linguistic complexity—primarily combinatorial in nature—triggered by combining stems and grammatical morphemes to form regular past tense verbs like *played* or *jumped* in English. A left hemisphere perisylvian network has been postulated to support key grammatical language functions both at the word level (Sahin et al. 2006; Bozic et al. 2010) and at the sentence level (Friederici 2012), whereas a complementary bihemispheric system is argued to support more general perceptual demands involved in the mapping from sound onto lexical meaning (Binder et al. 2000). Earlier fMRI and patient studies strongly support the contribution of left inferior frontal gyrus (left IFG); especially pars opercularis (BA44), in processing regular inflected forms (Ullman et al. 1997; Tyler et al. 2002, 2005; Bozic et al. 2010). Although previous findings suggest that left BA44 is recruited during morpho-phonological parsing that automatically segments the stem from the affix for regular inflected words (Tyler et al. 2005; Marslen-Wilson and Tyler 2007), the specific temporal dynamics of this process remain unknown.

This study investigates the neuronal basis of the linguistic and perceptual systems and tracks their interactions using functional cortical connectivity. Linguistically, complex words were regular inflected verbs (e.g., *played*), previously shown to trigger morpho-phonological parsing and activate the left frontotemporal system (Tyler et al. 2002, 2005; Bozic et al. 2010). Perceptual complexity is operationalized here in terms of lexical competition, as in previous studies (Bozic et al. 2010, 2013). Perceptually complex words (e.g., *claim*) have onset-embedded lexical competitors (e.g., *clay*) that trigger competition between multiple simultaneously active lexical candidates and increase load on the bilateral perceptual system (Bozic et al. 2010). Regular inflected words also have an onset-embedded stem (e.g., *play*) and potentially also load onto the bilateral system recruited during the processing of perceptually complex words, although this may be mitigated by the close morphological relationship between the stem and the full form. Both types of complex words were compared with simple words (e.g., *shape*) that were neither inflected nor contained an onset-embedded stem. A behavioral gating test was run to quantify the contrasts between items in terms of temporally varying levels of lexical competition. We then used a source reconstruction method to compute the evoked activity of the whole brain, based on EMEG responses of participants listening to words differing in their linguistic and perceptual complexity. Neural activity related to linguistic complexity showed a distributed network incorporating temporal cortices in both hemispheres and the frontal area BA44 in the left hemisphere. Tests of cross-communication between multiple brain regions showed that linguistically complex words generated specific cross-cortical communication in the gamma frequency band (20–60 Hz). This network only partially overlapped in space and time with the network supporting the processing of perceptually complex words. Finally, we showed that changes in functional connectivity between the left BA44 and posterior superior temporal gyrus (pSTG) areas are bound to the presence of linguistic complexity. Taken together, these results demonstrate that inflectionally complex words initiate morpho-phonological decomposition by engaging most strongly the perceptual and linguistic systems during the time window within which auditory input provides evidence for the grammatical morpheme. Noninvasive neuroimaging technologies reveal the transient frontotemporal assemblies that characterize the neural computations underpinning human speech comprehension.

## Materials and Methods

### Stimuli

The study used 240 words divided into 3 test conditions (80 words each): regular past tense (*played*), words with onset-embedded stem (*claim*), and simple words (*shape*) (see also Supplementary Table 1). All words were matched across conditions on word and lemma frequency, familiarity, and imageability (all *P* > 0.1, based on CELEX and MRC Psycholinguistic databases). The 240 test words were mixed with 160 fillers that were a mix of simple words (37%) and words with onset-embedded stems or suffixes (63%). The words were recorded in a sound-proof room by a female native speaker of British English onto a DAT recorder, digitized at a sampling rate of 22 kHz with 16-bit conversion, and stored as separate files using CoolEdit. Acoustic analyses were performed to compare the whole sound files across the 3 conditions (*played*, *claim*, and *shape*) with the dependent variables (intensity, pitch, and length) computed in Praat (http://www.fon.hum.uva.nl/praat/). The analyses of variance (ANOVAs) did not reveal any significant acoustical differences between them (all *P* > 0.20), with the duration ranging from 426 to 828 ms (mean ± SD, 593 ± 72 ms).

### Alignment Point

**Figure 1.**
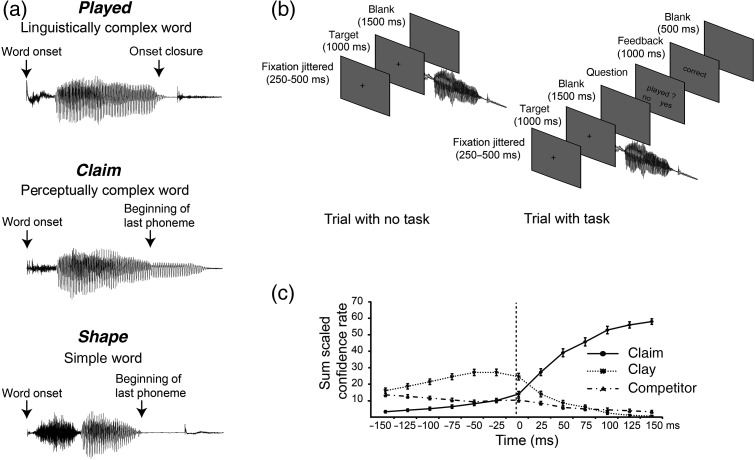
Alignment point, trial structure, and gating results. (*a*) Alignment point corresponding to the onset closure for regular inflected stimuli (*played*) and to the beginning of the last phoneme for the noninflected stimuli (*claim* and *shape*). (*b*) Representation of the trial structure with and without a task. (*c*) Gating results: Detailed pattern of responses (sum scaled confidence rate, see Supplementary Materials and Methods) for the onset-embedded words. The onset-embedded stem (e.g., *clay*) is the preferred answer before the alignment point, whereas the full form (e.g., *claim*) is the preferred answer afterwards.

For each item, an alignment point was identified individually (Fig. 1*a*). For the inflected words, this corresponded to the beginning of the silent period preceding the release of the final stop consonant (e.g., *d* in *played* or *t* in *walked*). We will refer to this as the *onset closure*. For monomorphemic words such as c*laim* and *shape*, we defined the alignment point as the timing corresponding to the beginning of their last phoneme. The last phoneme disambiguates the embedded stem *clay* from the actual target *claim*. Over 50% of the *claim* and *shape* stimuli ended in obstruent consonants (p/t/k/tch/g) similar to the *played* set (see Supplementary Table 1). We conducted a second set of acoustic analyses focused specifically around the pre-alignment and post-alignment periods separately (−200 to 0 ms and 0 to +200 ms). These showed lower intensity for linguistically complex items (*played*) compared with the other 2 sets in both periods: pre-alignment, *F*_2,237_ = 9.36, *P* = 0.00012 with *played* items being −2.64 db lower than *claim* items (*P* = 0.00004) and −1.92 db compared with *shape* items (*P* = 0.002); post-alignment, *F*_2,237_ = 12.22, *P* = 0.00001 with *played* items being −5.20 db compared with *claim* items (*P* = 0.000001), and −2.37 db compared with *shape* items (*P* = 0.02). No significant effect emerged for the pitch variable.

## Behavioral Gating Experiment

### Participants

Thirty-four native speakers of British English (mean age ± SD, 24 ± 6 years, range = 18–40) were recruited for the study. All had normal hearing, no known history of neurological problems, and normal or corrected-to-normal vision based on self-report. All gave informed consent and were paid for their participation. The study was approved by the Peterborough and Fenland Ethical Committee (UK).

### Stimuli

The 240 experimental stimuli were gated, with the construction of the gating materials controlled by the alignment point assigned to each item. We used gates 25 ms long: 6 gates before and 4 gates after the alignment point, covering a minimum of 250 ms acoustic information. The first segment always consisted of the word onset up to 150 ms before the alignment point (mean ± SEM, 400 ± 4.7 ms) plus the first gate (25 ms). For words with a silent period after the alignment point (all the inflected words and some words finishing with a voiceless consonant), we skipped the gates with no acoustic information, leaving on average 2.82 ± 0.06 gates per item after the alignment point (regularly inflected words: 2.02 ± 0.07, words with onset-embedded stems: 3.41 ± 0.10, and simple words: 3.05 ± 0.11). The total number of gates for each item varied from 7 to 10, depending on the length of the word in question. The last 2 ms of each segment was windowed to produce an accelerating attenuation eliminating audible clicks (Warren and Marslen-Wilson 1988).

### Procedure

We randomly assigned the 240 words to 6 lists, so that each testing session comprised 40 words (30 min). Each subject randomly completed 2 lists, with a 10-min break between sessions. The presentation and timing of stimuli was controlled using the Eprime software (www.pstnet.com). Gates from the same word were presented successively. Participants listened to the incomplete acoustic stimuli and were asked to type the first real word that came to mind. For each response, they were also asked to give their confidence rating on a 7-point scale (1: very confident and 7: not sure). Participants had no time pressure to give their answers. For each word and gate, we collected an average of 11.3 responses (minimum of 9). The test items were preceded by 7 practice items.

### Analyses

Data were first screened for nonword answers, which were rejected from further analyses. Errors in typing were corrected to an appropriate word answer when possible, or otherwise rejected. Confidence rates were reversed (answer 1 for confident was given a score of 7 and vice versa). For gates that were skipped after the alignment point, we entered the same answer (word and confidence rate) as the previous gate. All the data were normalized to 10 participants. For each gate and item, we computed the index “sum scaled confidence rate” which combines the number of participants giving that specific answer with their confidence rate. A maximum sum scaled confidence rate is 70 for 10 participants giving that specific answer and all participants being highly confident (7). Next, we computed a competition ratio (sum scaled confidence rate of competitor +1 divided by the sum scaled confidence rate of target +1) for each gate and type of word. Our focus was to determine whether the onset-embedded stem words (e.g., *claim*) elicited high levels of competition before the alignment point by biasing the percept of the participant towards the embedded stem (e.g., *clay*). We defined as competitors all responses except the embedded stem *clay*, which was defined as the target. For inflected words, the competitors included all responses except the stem *play* or its inflected form *played.* The target response incorporates *play* and *played.* For simple items, the competitors include all responses except the full form *shape*. The higher the competition ratio, the more competition there is for the specific target. The resulting competition ratios were entered into a two-way ANOVA with gates (13) and word types (3: *played*, *claim*, and *shape*) as fixed effects. To unpack the interaction, a competition ratio was averaged within 3 different time windows, from −150 to −100 ms, from −75 to 0 ms, and from +25 to 100 ms post-alignment point and entered into separate ANOVAs with word type as a fixed effect (see Supplementary Fig. 1).

## EMEG Experiment

### Participants

Twenty-two right-handed native speakers of British English (who did not participate in the behavioral gating experiment) were recruited for the study. All had normal hearing, no known history of neurological problems, and normal or corrected-to-normal vision based on self-report. All gave informed consent and were paid for their participation. The study was approved by the Peterborough and Fenland Ethical Committee (UK). Five participants were rejected from the analyses (3 because of a technical problem during the acquisition and 2 because of too many blink artifacts, see below for description), leaving a sample size of 17 (7 men, mean age ± SD, 25 ± 4 years, range = 19–35).

### Procedure

Each trial consisted of a centrally presented fixation cross with length jittered between 250 and 500 ms (Fig. 1*b*). While the cross stayed on for another 1000 ms, an auditory word was presented, followed by a blank screen for 1500 ms. For 8% of the trials, a probe was presented after the blank screen with a written word. The task of the participant was to indicate whether the word matched the preceding acoustic stimulus or not (one-back memory). Half of the participants answered “yes” with the right hand and “no” with the left hand. The other half used the reverse combination. Feedback was presented on the screen for 1000 ms and followed by a blank screen of 500 ms. The presentation and timing of stimuli was controlled using the Eprime software (www.pstnet.com). The stimuli were binaurally presented at approximately 65 dB SPL via nonmagnetic earpieces. Each item was presented twice in a pseudorandom order across 7 blocks of stimulation (6 min each). Each participant received 20 practice trials, which included a presentation of each different stimulus type and 3 exemplars of one-back memory trials.

### EMEG Recording

Continuous MEG data were recorded using a VectorView system (Elekta-Neuromag, Helsinki, Finland) containing 102 identical sensor triplets, composed of 2 orthogonal planar gradiometers and one magnetometer, covering the entire head of the subject. Participants sat in a dimly lit magnetically shielded room (IMEDCO AG, Switzerland). The position of the head relative to the sensor array was monitored continuously by feeding sinusoidal currents into 4 head position indicator (HPI) coils attached to the scalp. The simultaneous EEG was recorded from 70 Ag–AgCl electrodes placed within an elastic cap (EASYCAP GmbH, Herrsching-Breitbrunn, Germany) according to the extended 10/20 system and using a nose electrode as the recording reference. Vertical and horizontal electrooculograms (EOG) were also recorded. All data were sampled at 1 kHz with a band-pass filter from 0.03 to 330 Hz. A 3D digitizer (Fastrak Polhemus, Inc., Colchester, VA, USA) was used to record the locations of the EEG electrodes, the HPI coils, and approximately 50–100 “headpoints” along the scalp, relative to three anatomical fiducials (the nasion and left and right preauricular points).

### Data Preprocessing

Static MEG bad channels were detected and excluded from all subsequent analyses (MaxFilter; Elekta-Neuromag). Compensation for head movements (measured by HPI coils every 200 ms) and a temporal extension of the signal space separation technique (Taulu et al. 2005) was applied to the MEG data (MaxFilter; Elekta-Neuromag). Static EEG bad channels were visually detected and interpolated (Hämäläinen and Ilmoniemi 1994). The EEG data were re-referenced to the average over all channels. The continuous data were low-pass filtered to 30 Hz and epoched with respect to the alignment point (onset closure for inflected words and beginning of the last phoneme for noninflected words). Epochs included the 200 ms before to 200 ms after the alignment point. Baseline correction was applied by subtracting the average response of the 100 ms prior to the 200 ms time window from all data points throughout the epoch. Trials were rejected based on eye movement or blink artifacts detected by EEG/EOG (>200 μV), or high magnetometer (>4000 fT) or gradiometer (>2000 fT/cm) values (leaving 92 *played* epochs, 94 *claim* epochs, and 93 *shape* epochs on average across participants). Visual inspection of the waveforms at the sensor level (see Supplementary Fig. 3) showed the importance of the realignment procedure compared with the word onset alignment for revealing differences between our conditions (e.g., Leminen et al. 2011).

### Source Reconstruction

The location of the cortical current sources cannot be precisely determined using the measured magnetic fields from outside the head. Here, we estimate the location of these sources using the neuroanatomically constrained minimum norm estimate (MNE) procedure, based on distributed source modeling rather than equivalent current dipoles (Hämäläinen et al. 1993). Since most of the electromagnetic signals originate from postsynaptic currents in the apical dendrites of cortical pyramidal cells in the cortex, the orientation of these currents is tangential to the cortical mantle. MNE therefore computes the inverse solution taking into account the individual anatomical information provided by a structural magnetic resonance imaging (MRI) scan of each participant, using the boundary between gray and white matter for its algorithm. A complete overview of the MNE suite for EMEG source estimation can be found in Gramfort et al. (2014). For each participant, MRI images were obtained using a GRAPPA 3D magnetization prepared rapid acquisition gradient echo (MPRAGE) T1-weighted scans (time repetition = 2250 ms; time echo = 2.99 ms; flip angle = 9°; acceleration factor = 2) on a 3-T Trio (Siemens, Erlangen, Germany) with 1 mm isotropic voxels. From the MRI data, a representation of the cerebral cortex was constructed using the FreeSurfer program (http://surfer.nmr.mgh.harvard.edu/) to separate the scalp, skull, and brain. The forward model was calculated with a three-layer boundary element model using the outer surface of the scalp as well as the outer and inner surfaces of the skull identified in the anatomical MRI with different electrical conductivities for each compartment (Gramfort et al. 2014). The interface between the gray and white matter representation was downsampled to yield a source space of 10 242 vertices per hemisphere that was used as the location of the dipoles (average spacing of 3.1 mm between dipoles). Anatomically constrained activation movies were created by combining MRI, MEG, and EEG data, providing a better source localization than MEG or EEG does independently (Liu et al. 2002). Fusing all modalities increases the conditional precision of the underlying source estimates relative to that obtained by inverting magnetometers, gradiometers, or EEG alone (Henson et al. 2009). To visualize activation across subjects, the cortical surface representations of individual subjects were aligned using a spherical morphing technique (Fischl, Sereno, Tootell, et al. 1999) and inflated (Fischl, Sereno, Dale 1999). We employed depth weighting to correct for MNE's bias toward attributing signals to superficial sources and a loose-orientation constraint (0.2), as recommended by Lin et al. (2006), to improve the spatial accuracy of localization. Sensitivity to neural sources was improved by calculating a noise covariance matrix based on the 100-ms prestimulus period. The activations at each location of the cortical surface were estimated over 1 ms windows, resulting in spatiotemporal brain activation movies.

### Analyses

#### 3D (Space × Time) Sensor SPM

The sensor-level analysis was performed on each sensor type separately, since they reveal different features of the underlying generators, and since each forms a separate input to the MNE source reconstruction process. The magnetometers and the gradiometers have intrinsically different noise levels, with magnetometers linked to a better detection of deeper sources compared with gradiometers (Henson et al. 2009). EEG is sensitive to different orientation of the neuronal currents (radial and tangential), thus providing complementary information (Baillet et al. 1999; Liu et al. 2002; Sharon et al. 2007; Molins et al. 2008). We performed a mass univariate analysis using Statistical Parametric Map (SPM) 5 (www.fil.ion.ucl.ac.uk/spm/), in which *F*-tests were computed at every point in a 3D image of channel Space × Time. These analyses (and all those reported below) were conducted on the data aligned to the onset closure alignment point (Fig. 1*a*). The topographic distribution of each sensor type was transformed into a 2D space by linear interpolation to a 32 × 32 pixel grid, with the time dimension consisting of 501 time samples (1 ms each) in the epoch (including the baseline). *F*-tests corresponding to the main effect of condition (*played*, *claim*, and *shape*) were performed and thresholded at a voxel level of *P* < 0.001, and at *P* < 0.05 for extent using the nonstationarity toolbox (Hayasaka et al. 2004).

#### Cortical Regions-of-Interest Analysis

The first set of source space analyses was conducted on a set of a priori anatomically defined regions of interest (ROIs) based on the group average inflated cortex produced by FreeSurfer. We focused on a set of bilateral frontotemporal ROIs incorporating frontal and temporal areas [BA44, BA45, BA47, Heschl's gyrus (HG), posterior superior temporal sylcus (pSTS), supramarginal gyrus (SMG), and superior, middle, and inferior temporal gyri (STG, MTG, ITG)]. Temporal gyri were divided into anterior and posterior ROIs, creating a total of 12 ROIs per hemisphere (see Supplementary Fig. 2]. The activation time course (−200 to +200 ms relative to the onset closure alignment point) of each condition was extracted and averaged across all vertices within each ROI. The current for each subject was calculated every 1 ms, baseline-corrected (using the pre-epoch period from −300 to −200 ms), and statistically compared using paired-sample *t-*tests at every time point (separate analyses for *played* vs. *shape* and *claim* vs. *shape*). To control for multiple comparisons, cluster-mass permutation corrections were used (Maris and Oostenveld 2007). This method calculates the cluster size of an effect (the number of contiguous significant effects, here in the temporal domain) that exceeds the alpha level using 10 000 permutations of the data (one-sided; *P* < 0.05). To assess the specificity of the significant clusters, a follow-up analysis evaluated the extent to which they differ from the activity triggered by the remaining condition. This was done by computing the mean value for the 3 experimental conditions over the whole period of each cluster and then computing a *t*-test for dependent samples to evaluate potential differences in the level of activity between *played* versus *claim* and *claim* versus *shape* separately. Finally, a laterality index was computed to characterize the frontotemporal network of each type of word. Amplitude of source activity for left and right hemispheres was averaged over the 12 ROIs for 2 specific time windows: pre-alignment (−200 to 0 ms) and post-alignment (0 to +200 ms). The laterality index (LH − RH)/(LH + RH) was subjected to repeated-measures ANOVAs with factors condition (*played*, *claim*, and *shape*) and time window (pre-alignment and post-alignment). Positive values indicate left-lateralized activity across frontotemporal ROIs.

#### Cortical Phase-Locking Analysis

To reveal the dynamics of the neural processes while linguistic information unfolds within the incoming speech, we computed intertrial phase-locking values (PLVs). We tested all potential synchronies between the 8 ROIs that showed a significant increase of activity for inflected items (Fig. 3*a*) and the rest of the bilateral frontotemporal network (23 ROIs; see also Supplementary Fig. 2). For example, left pSTG was tested against the 23 other ROIs, 11 on the left, and 12 ROIs on the right hemisphere. Trial-by-trial phase-locking between neural brain areas (Lachaux et al. 1999) was determined by mapping each ROI onto the individual subjects' cortical surface. A single time series was extracted for each ROI by projecting the continuous data onto the cortical surface using the inverse solution operator (computed during the MNE source reconstruction). The single-trial data were then epoched (−300 to 600 ms) and baseline-corrected (−300 to −200 ms). To avoid any phase-locking due to coincidental overlap of evoked responses between 2 regions, the average-evoked response was subtracted from each trial (leaving only the induced phase-locking). A signal phase angle was obtained for each time point and frequency of interest (10–60 Hz, 1 Hz step) by filtering the data with an Morlet wavelet decomposition (factor 7). The PLV between 2 regions was computed (Lachaux et al. 1999) and baseline-corrected (−300 to −200 ms). PLVs range from 0 (random) to 1 (aligned) and are inversely correlated with the variance in the trial-by-trial phase difference between 2 signals (see Supplementary Fig. 6). The PLV value therefore reflects the synchrony between 2 signals tested. PLV differences between conditions were statistically determined using paired-samples *t*-tests. Cluster-mass permutation tests (10 000 permutations) with an alpha level of 0.05 (one-tailed) was used to determine the significance of each cluster and to control for multiple comparisons (Maris and Oostenveld 2007). Finally, we extracted the gamma-band PLV values during the time window defined in the previous analysis for each significant cluster, condition, and subject. We assessed the specificity of each cluster with a *t*-test for dependent samples to evaluate potential gamma phase synchrony differences between *played* versus *claim* and *claim* versus *shape* separately. Latency analyses were computed by searching for the maximum differences in the PLV values between complex words and simple words and tested using the sample *t*-test.

## Results

### Behavioral Gating Task

To document the degree of competition for the perceptually complex (onset embedded) *claim* words relative to the *played* and *shape* set, and to specify the potential timing of these competition effects, we carried out a gating experiment where the listeners heard successively larger fragments of the stimulus and were asked to guess which word they thought they were hearing. The results revealed a modulation of the level of competition depending on word type (*F*_2,237_ = 238.91, *P* < 0.001) and gate (*F*_12,2844_ = 102.91, *P* < 0.001), with a significant word type × gate interaction (*F*_24,2844_ = 163.78, *P* < 0.001; Fig. 1*c* and see Supplementary Fig. 1). Post hoc analyses showed that, between −150 and −100 ms before onset closure, both types of complex words have a lower level of competition compared with the simple words (*F*_2,237_ = 17.938, *P* < 0.001). For the perceptually complex words (*claim*), listeners are predominantly choosing the wrong stem *clay* instead of *claim*. Between −75 and 0 ms before closure, inflected words showed less competition compared with the 2 other types of words (*F*_2,237_ = 7.35, *P* < 0.001). For the embedded stem (*claim*) sets, the level of competition increases due to the accumulation of acoustic cues that *clay* is not the target but *claim*. Inflected words at this stage have their stem (*play*) fully selected and recognized. After the alignment point (+25 and 100 ms), the *clay* stem is no longer the preferred answer so that the *claim* set has a markedly higher competition level compared with the inflected and simple words (*F*_2,237_ = 200.06, *P* < 0.001). These results confirm that the *claim* sets differ markedly from the *played* and *shape* sets in the level of lexical competition—and therefore in potential perceptual complexity—at the critical time points aligned around the onset closure.

### EMEG Sensor-Level Analysis

**Figure 2.**
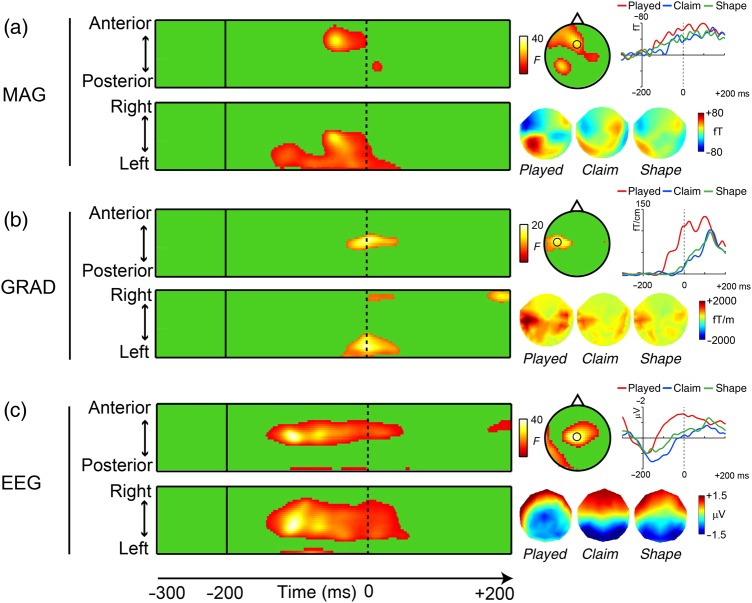
SPM sensor level. Left: Two-tailed space–time SPMs for the significant main effect of condition for magnetometers (MAG, *a*), gradiometers (GRAD, *b*), and EEG (EEG, *c*). The 2 images in each panel represent orthogonal planes (*x*-*t*, *y*-*t*) through the 3D image at the location of the main significant clusters. For each type of sensors, another smaller cluster also reached significance (see Supplementary Table 2). Right: Mean distribution of each cluster at peak latency, with an evoked waveform from the sensor showing the maximal difference (black circle on topographies). The gradiometer topography and waveform reflect the root mean square value across the 2 planes, orthogonal gradiometers. The mean sensor-level topographies for each condition, averaged over time for each significant cluster (MAG: [−130 to +30 ms], GRAD [−30 to +35 ms], and EEG [−145 to +45 ms]) and across participants, are represented underneath.

Analysis of the EMEG data at the sensor level suggests that, compared with perceptually complex words (*claim*) and simple words (*shape*), linguistically complex words (*played*) increased activity across all 3 types of sensors (EEG, magnetometers, and gradiometers; Fig. 2). These effects started around 145 ms before the onset closure, continuing to 45 ms after the closure (see Supplementary Table 2). The behavioral gating test (see Supplementary Fig. 1*b*) showed that this corresponds to the time when acoustic input was sufficient to reveal the lexical identity of the stem (−150 to 0 ms pre-onset closure) and the presence of a grammatical suffix (0–100 ms Post-onset closure). Most of the activation clusters showed a left-lateralized distribution, with frontal, temporal, or posterior maxima (Fig. 2, right), suggesting a greater involvement of left hemisphere cortical generators related to processing linguistic complexity.

### EMEG Source-Level Analysis

**Figure 3.**
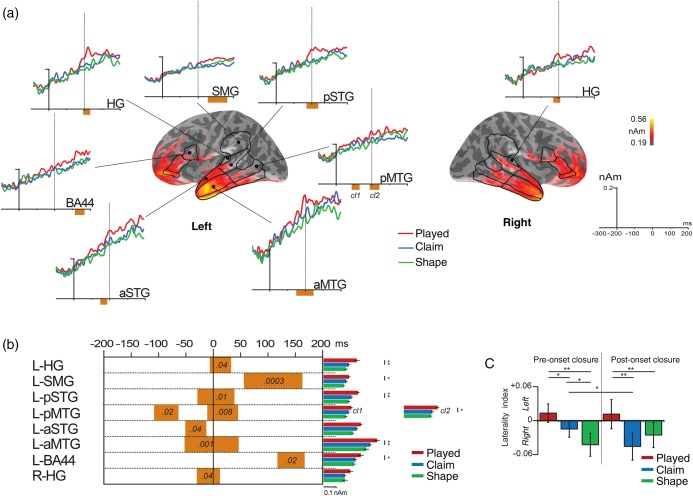
The cortical activity supporting the comprehension of a regular inflected word (*played*). (*a*) The mean current distribution (minimum norm source estimates) for the linguistically complex *played* words, averaged during the time window of the analysis (−200 to +200 ms post-onset closure). Line plots show the mean time courses for the significantly activated ROIs with cluster permutation analysis (*played* vs. *shape*): red—*played*, blue—*claim*, and green—*shape* words. The ROIs are visualized by black outlines. The orange squares indicate a significant cluster (*P* < 0.05, corrected for multiple comparisons). (*b*) Details of the significant clusters. Each cluster is represented by a square with the length corresponding to its timing. *P-*values are indicated inside the box. On the right side are plots of the mean amplitude of the neural activity for each condition within the significant cluster. We report significant *t*-test (*played* vs. *claim*; *claim* vs. *shape*) with **P ≤ 0.01 and *P < 0.05. (*c*) Laterality index (LH − RH)/(LH + RH) of the activation, with positive values suggesting left-lateralized activity in the frontotemporal regions. Those values have been entered into an ANOVA with factors condition (*played*, *claim*, and *shape*) and time window (pre-onset closure and post-onset closure). Significant post hoc analyses are coded with **P < 0.01 and *P < 0.05.

Whole-brain source reconstructions showed a range of frontal, temporal, and parietal bilateral areas activated for the 3 types of words over the 400-ms onset closure-aligned epoch. This is characteristic of the neural activation for auditory language processing (Marinkovic et al. 2004; see Supplementary Fig. 4). Linguistically complex words (*played*) enhanced activity compared with simple words (*shape*) in the left perisylvian network, including 6 temporal/parietal areas [pSTG, HG, posterior middle temporal gyrus (pMTG), SMG, anterior middle temporal gyrus (aMTG) and anterior superior temporal gyrus (aSTG)], 1 frontal area (BA44), and 1 brain area on the right (HG; Fig. 3*a*). All clusters started before the onset closure, except for L-SMG (+57 ms) and L-BA44 (+119 ms; Fig. 3*b*). Perceptually complex words (*claim*) did not substantially differ from simple words (Fig. 3*a* and see Supplementary Fig. 4). Moreover, the results showed that the increased activations in left HG, SMG, pSTG, pMTG (*cl2*), aMTG, and BA44 are specific to the presence of a linguistic grammatical morpheme (all *P* < 0.05, Fig. 3*b*, right and see Supplementary Table 3). All of this linguistically specific activity occurred around the onset closure, during the period when the acoustic input signals the potential presence of an inflectional morpheme. Lateralization of activity across the whole frontotemporal network revealed a significant effect of condition (*F*_2,32_ = 3.38, *P* < 0.05) and a significant interaction between condition and time window (*F*_2,32_ = 3.25, *P* = 0.05). Consistent with previous results, post hoc tests showed that inflected words elicited stronger left-lateralized activity before and after the onset closure compared with the other types of words (Fig. 3*c*). Words that were perceptually rather than linguistically complex, due to competition from an embedded stem (*clay/claim*), showed stronger right lateralized activity after the onset closure.

### Phase-Locking Analysis

**Figure 4.**
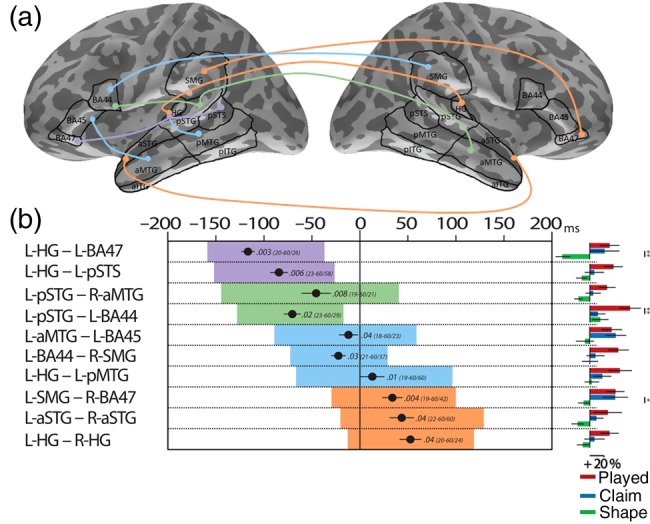
Patterns of phase synchrony for linguistically complex words in the gamma range (20–60 Hz). (*a*) Phase-locking analysis showing significant trial-by-trial phase covariance between ROIs for the linguistically complex words (*played*) compared with simple words (*shape*). All the synchronies involved the gamma band between 20 and 60 Hz. Colors code the start of the synchronies: purple between −200 and 150 ms, green between −150 and −100 ms, blue between −100 and −50 ms, and orange after −50 ms. No synchrony starts after the onset closure. (*b*) Details of the significant synchronies. Each significant cluster is represented by a box whose length corresponds to its timing. *P*-values (*P* < 0.05, corrected for multiple comparisons) and frequency band (min–max/peak in Hz) are indicated inside the box, color-coded as previously. The average latency of maximum difference between the linguistically complex word (*played*) and simple word (*shape*) is reported (mean: black circle; ±SEM: horizontal bar). On the right side are plots of the mean percentage change in PLV relative to the baseline for each condition within each significant cluster. We report significant *t*-test (*played* vs. *claim*; *claim* vs. *shape*) with **P ≤ 0.01 and *P < 0.05.

We next investigated how the brain areas involved in linguistically complex computation interact with each other, by defining their functional connectivity. The network for processing linguistic complexity involved synchronies in 10 ROIs on the left and 5 on the right hemisphere, from 160 ms pre-onset closure to 129 ms post-onset closure (Fig. 4). The oscillations covered frequencies between 20 and 60 Hz, incorporating beta and (predominantly) gamma bands. For simplicity, we will refer to 20–60 Hz as a gamma band in the following sections. For the left hemisphere synchronies, 3 involved long-distance frontotemporal interactions and 2 were between temporal areas (Fig. 4*a*). Using the number of interactions as a criterion, we identified 3 brain regions (L-HG, L-pSTG, and L-BA44) as central network nodes interacting with all other brain areas. Since some synchronies from these central network nodes overlapped in time (Fig. 4*b*), we further investigated these temporal differences. L-HG connects first with frontal L-BA47 (−117 ms before the onset closure) and then with L-pSTS (−84 ms; *t*_(1,16)_ = −3.3, *P* < 0.003). The remaining 2 L-HG synchronies occurred later: with L-pMTG at +12 ms and with R-HG at +52 ms (all *P* < 0.0001). The L-pSTG node synchronized simultaneously with R-aMTG and L-BA44 (−47 and −71 ms, respectively; *P* > 0.15). Finally, L-BA44 synchronized earlier with L-pSTG (−71 ms) than with R-SMG (−23 ms; *t*_(1,16)_ = −5.3, *P* < 0.0001).

### Specificity of the Linguistic Complexity Oscillations

**Figure 5.**
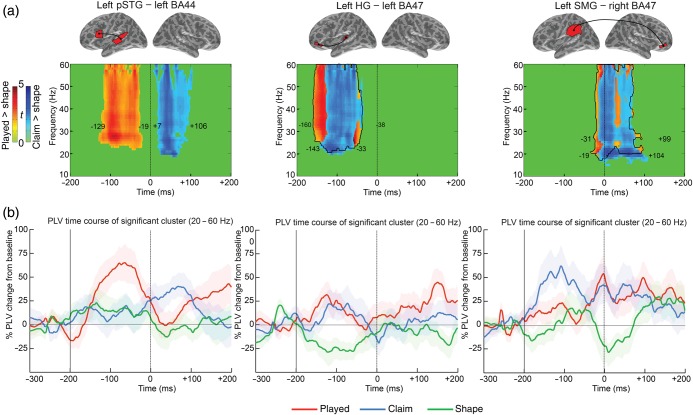
Time course analysis of the synchrony involving common ROIs between both complex word-types. (*a*) Time by frequency clusters of significant PLV differences between linguistically complex words (*played*) and perceptually complex words (*claim*) versus simple words (*shape*) (*P* < 0.05 corrected for multiple comparisons). Color reflects absolute *t-*values for the *played* vs. *shape* trials (red cluster) and for *claim* vs. *shape* trials (blue cluster; only significant *t-*values are shown). Left: left pSTG–left BA44; middle: left HG–left BA47; right: left SMG–right BA47 with the dark line corresponding to the limit of the red cluster. (*b*) Percentage change from baseline ± SEM time course of PLV in the gamma band for each synchrony. Left: phase synchrony between left pSTG and left BA44 was enhanced in the gamma band (20–60 Hz) from −129 to −19 ms pre-onset closure for *played* words relative to items that do not have a suffix (*claim* and *shape*). Middle: phase synchrony between left HG and left BA47 was enhanced in the gamma band (20–60 Hz) for both complex words (*played* and *claim*) relative to simple words (*shape*). Timing overlapped largely with a cluster from −160 to −38 ms for *played* and from −143 to −33 ms for *claim*. Right: phase synchrony between left SMG and right BA47 ROIs. Both complex words increased significantly differential phase synchrony concentrated in the gamma frequency band (20–60 Hz) with a similar timing: from −31 to +99 ms for *played* and from −19 to +104 ms for *claim*.

To determine whether increased processing demands due to linguistic and perceptual complexity shared processes within the perisylvian network, we directly compared the PLVs for the 3 types of words within each significant cluster obtained from the *played* versus *shape* contrast. [We also tested separately the perceptual network (*claim* vs. *shape*) and results showed a limited numbers of synchronies with only partial overlap with the linguistically subsystem (see also Supplementary Fig. 5).] The results showed that 2 synchronies (L-HG–L-BA47 and L-SMG–R-BA47) are shared between the 2 types of processes and 1 (L-pSTG–L-BA44) is specific to the linguistically complex words (Fig. 4*B*, right and see Supplementary Table 4). Between −129 and −19 ms, the L-pSTG–L-BA44 gamma oscillation is highly specific to linguistically complex words (+55%) compared with both *claim* (+11.1%, *P* < 0.01) and *shape* words (+14.3%, *P* < 0.02; Figs 4*B*, right and 5, left; see Supplementary Table 4). Later on, between +7 and +106 ms, the synchrony is induced by the perceptually complex words (*claim*) compared with *shape* items (*P* < 0.04; see Supplementary Fig. 5), but does not differ from the inflected words (Fig. 5*b*, left). In contrast, the synchrony L-HG–L-BA47 emerged for both types of complex words (*played*, *P* < 0.003 and *claim*, *P* < 0.02), overlapping in frequency band (gamma 20–60 Hz), and timing (−160 to −38 ms for inflected and −143 to −33 ms for onset-embedded words; Figs 4*B*, right and 5*a*, middle). The effect held equally for both linguistically (+17%) and perceptually complex words (+13%) compared with simple words (−24%; Fig. 5*b*, middle). Similar results held for the L-SMG–R-BA47 synchrony with a significantly greater gamma phase between −31 and +99 ms for inflected words (*P* < 0.004; +35%) compared with *shape* (−8%), but no difference between inflected words and onset-embedded *claim* words (+34%; *P* > 0.10; Fig. 5, right). [Similar results emerge when analyses are performed for the second timing reported in the PLV analysis, comparing *claim* vs. *shape* (between −19 and +104 ms).] Finally, the gamma L-pSTG–L-BA44 oscillation which is specific to the processing of an inflected ending starts later (−71 ms pre-onset closure) compared with L-HG–L-BA47 for both complex words (*played*: −117 ms, *t*_(1,16)_ = 4.63, *P* < 0.0002; *claim*: −98 ms, *t*_(1,16)_ = 3.05, *P* < 0.007). Inflected words exhibit an earlier latency than the embedded words for the L-HG–L-BA47 synchrony (*P* = 0.057).

## Discussion

We characterized the dynamic neural machinery engaged in the combinatorial analysis of linguistically complex words like *played*, consisting of stems and inflectional suffixes. Linguistic complexity modulated activity on the scalp surface at time points where the acoustic input signaled the presence of a suffix (Fig. 2), and source reconstruction consistently localized this activity to left perisylvian areas (Fig. 3). Further analyses suggested that the processing of linguistically complex words is mediated by the temporary formation of 2 dynamic ensembles that evaluate different types of information. First, there is a rapid identification of relations between the phonological elements, reflecting the perceptual analysis that leads to lexical access of the stem. This computation involves left anterior and posterior temporal cortices (Fig. 3) and connects to BA47 bilaterally (Fig. 4). Secondly, there is integration between lexical and grammatical units, within a larger percept structured according to abstract regularities (stem + suffix). This is supported by the linguistic subsystem, recruiting left BA44 and temporal areas (Fig. 3). This left perisylvian network is coordinated via gamma-band frequency oscillations, which bind left temporal and frontal areas more intensely during morpho-phonological parsing (Fig. 4). Taken together, the results show that extra processes are required for recognizing an inflected word compared with simple words, which first reflect the recognition of the stem, and then its integration to a larger structure with the suffix (Marslen-Wilson and Tyler 2007; Tyler and Marslen-Wilson 2008). They support the notion that language comprehension needs a flexible and interactive dual system, with the processing of linguistically complex words engaging both perceptual and linguistic processing mechanisms.

### Linguistic Versus Low-Level Effects

A necessary consideration, given the acoustic differences between the *played* set and the *claim* and *shape* sets at onset closure, is to determine whether the increased activity for linguistically complex words observed at both sensor and source levels might be related to low-level acoustic differences in our stimuli. In terms of overall loudness, acoustic analyses of the pre-onset and post-onset periods separately (−200 to 0 ms and 0 to +200 ms) suggest that, in both periods, linguistically complex items (*played*) showed lower intensity compared with the other item types. Several studies of brain activity accompanying changes in intensity with either tone bursts or continuous stimuli have shown that response amplitude increases and latency decreases with increasing intensity (Pantev et al. 1989; Dimitrijevic et al. 2009). The source intensity of the auditory response in the vicinity of Heschl's gyrus/planum temporale (PT) also increases with the amplitude of acoustic input. Here, we see a reverse effect, such that morphologically complex words (*played*), despite their overall lower intensity, elicit larger amplitude responses at the sensor and source level, suggesting that these low-level acoustic differences cannot account for our results.

Another potential difference, however (although shared with over 50% of the *claim* and *shape* sets), is that *played* items inherently contain a silent (or at least reduced energy) period after the alignment point, followed by a release of the plosive component of the word-final obstruent (Fig. 1*a*). These onsets and offset may trigger transient EMEG off- and on-responses, leading to sharp but short-lived increases in the signal. In the MEG literature (e.g., Pantev et al. 1996; Yamashiro et al. 2011) on- and off-responses, elicited by the abrupt onset or offset of a continuous tone, elicit an N1-type waveform at latencies of 80–100 ms after acoustic onset/offset, and localized in pSTG or HG bilaterally. While we cannot rule out some contribution of off-responses to the observed response curves (on-responses to the stop releases, if present, would have fallen largely outside our epoch of interest), we would expect to see these emerging only after onset closure, at 80–100 ms delays. This is not the dominant pattern seen here, where the significant increase in neural response for inflected words starts up to 150 ms before the alignment point (Fig. 2), where this is not a transient peak like the N1 but a sustained activity continuing well beyond closure (Fig. 2 and see Supplementary Fig. 3), and where the increase is not simply seen in pSTG and HG and is left lateralized (Fig. 3; see Supplementary Figs 3 and 4). This spatiotemporal patterning of the *played* effects strongly suggests that these reflect linguistic rather than acoustic properties of the stimuli. The exact contribution of acoustic transients should be looked at, however, in future studies focused on this point.

### Core Regions for Processing Linguistically Complex Words

The involvement of left temporal areas for processing inflected words has been commonly reported in MEG studies and related to lexical access of the stem (Vartiainen et al. 2009; Leminen et al. 2011); no previous MEG research showed frontal involvement in such processing (fMRI studies of inflected forms reported the involvement of both sets of brain areas, for a review see Tyler and Marslen-Wilson 2008). The presence of frontal effects in the current study supports the claim that EEG measurements can supplement the information provided by MEG alone (Baillet et al. 1999; Liu et al. 2002; Sharon et al. 2007; Molins et al. 2008). Both anterior and posterior temporal areas were implicated, with potentially different functional roles. Left anterior temporal brain areas (aSTG/aMTG) enhanced activity as soon as the stem was heard and recognized, arguably reflecting lexical selection of the stem (Scott and Johnsrude 2003; DeWitt and Rauschecker 2011; Fig. 1*c* and see Supplementary Fig. 1). Left posterior temporal areas (HG and pSTG) were selectively activated during the onset closure period, when the unfolding acoustic input signaled the presence of a potential grammatical morpheme. Left pSTG and surrounding areas (HG and PT) have been shown to react to speech processing even in the absence of acoustic input (imagery or silent speech; Price 2012). The left pSTG (incorporating PT) may act as a “hub” by constructing a transient representation of the spectrotemporal structures of the grammatical morpheme that reflect the (automatic) computation of regular sound sequences and the prediction of future auditory events (Griffiths and Warren 2002). Left pMTG, which also showed increased activity for linguistically complex words at this time, has been related to mapping phonological information into stored meaning representations and retrieving lexicosyntactic information from memory (Scott and Johnsrude 2003; Hickok and Poeppel 2007; DeWitt and Rauschecker 2011; Friederici 2012; Price 2012; Tyler et al. 2013). Left SMG activated after onset closure may be related to the activation of abstract phonological units with a crucial role in the phonological working memory (Jacquemot et al. 2003). Finally, and post-onset closure, the posterior left IFG BA44 was sensitive to the phonological cues that signal morphological decomposition. This timing corresponds to the release of the suffix, and we suggest that the increased activation of left BA44 at this time reflects the integration of the affix with the previously recognized stem (Hagoort 2005).

### Gamma Oscillations and the Language System

Long-distance cortico-cortical coupling revealed a network of oscillations in the gamma frequency band (20–60 Hz). Long-range synchronizations occur when 2 large neuronal populations located at 2 distant locations oscillate with a similar phase over a few cycles and enable communication between these distant brain areas (Fries 2005). This is in contrast to local synchronizations which are likely to occur when a large number of neurons oscillate with a common phase at a specific location. Thus, the local synchronization as measured by power analysis is thought to reflect local networks (within a node of a functional network), whereas long-range synchronization measured by PLV analysis indicates the formation of functional long-range networks (between different nodes of a network; Bastiaansen and Hagoort 2006). As a fast rhythm, gamma oscillation is well suited to cognitive and language processing because of its ability to quickly form transient networks and plays an important role in binding spatial and temporal information in different brain areas to build a coherent percept (Varela et al. 2001). Interestingly, within the left hemisphere, our frontal–temporal long-range connectivities have a peak frequency oscillating in the beta range (L-pSTG–L-BA44: 29 Hz; L-HG–L-BA47: 26 Hz; L-aMTG–L-BA45: 23 Hz, Fig. 4*B*), whereas the temporal–temporal connectivities exhibit higher frequency oscillations (L-HG–L-pSTS: 58 Hz; L-HG–L-pMTG: 60 Hz). These results are consistent with the proposed hypothesis that faster rhythms like gamma are well suited to the scale of local synchronization (mm–cm), while lower frequencies are better suited to long-range communications since they typically synchronize more slowly (Kopell et al. 2000; von Stein and Sarnthein 2000). Nonetheless, recent work shows that long-range synchronizations can also occur at substantially higher frequencies (>30 Hz) in cats (Engel et al. 1991), monkeys (Buschman and Miller 2007; Gregoriou et al. 2009), and also in humans (Rodriguez et al. 1999; Hipp et al. 2011; Palva and Palva 2012). Gamma oscillations could also be a suitable frequency channel for the brain to communicate at long-range distances. An alternative interpretation suggests a different functional basis to the dissociation between beta- and gamma-band long-range oscillations, where feed-forward bottom-up information is propagated on gamma frequency channels, whereas recurrent top-down processes primarily use beta frequency channels (Fries 2009; Arnal et al. 2011). Both hypotheses fit our data and further research will be necessary to distinguish between these interpretations.

Most of the previous studies investigating language processing have focused on local synchrony (power spectral changes) while ignoring the relationship between different brain areas (long-range synchrony). In spoken word comprehension, fast oscillation (gamma) is adequate to capture transient broadband bursts of energy and fast formant transitions (Rosen 1992) and may represent the speech input at the phoneme level (Giraud and Poeppel 2012). An increase in local gamma oscillations have been linked to various levels of language comprehension, from perceptual processing (Pantev et al. 1991), lexical access (Pulvermüller et al. 1996; Hannemann et al. 2007; Tavabi et al. 2011), semantic integration (Hagoort et al. 2004; Hald et al. 2006; Penolazzi et al. 2009) to phonological encoding (Wheat et al. 2010). Relevant to this, Mainy et al. (2008) distinguished between local gamma oscillation networks located in B44 for phonological processing and in BA45/47 for semantic processing. Intracranial recordings (iEEG) and electrocorticography also report modulation of gamma oscillation related to language processing, although in higher spectral bands (70–200 Hz) (Crone et al. 2001; Mainy et al. 2008; Jerbi et al. 2009).

Long-range gamma oscillations have been found during language processing with scalp EEG (Ford et al. 2005; Reiterer et al. 2011; Molinaro et al. 2013), intracranial recordings (Crone et al. 2006; Chen et al. 2011), and MNE source reconstruction (Gow et al. 2008; Han et al. 2012). All of these studies support a role for gamma-band oscillations in spoken language comprehension with a strong interaction between temporal and frontal areas (Ford et al. 2005). Long-range gamma neural synchrony is linked with performances in reading (Han et al. 2012) and also with proficiency in second-language speakers (Reiterer et al. 2011). It is therefore not surprising that our long-range synchronies fall into the fast frequencies, consistent with the view that gamma oscillations are an important part of the functional network that subserves normal spoken comprehension (Hald et al. 2006).

Our results further suggest a hub-like structure in which left HG, pSTG, and BA44 are key nodes and synchronized most prominently with other nodes of the gamma network (Fig. 4). The evoked analysis (Fig. 3) revealed only a modulation of activity in left BA44, whereas the induced analysis of functional synchrony (Figs 4 and 5) showed the involvement of both left and right BA47 and left BA44 in the processing of inflected words. Induced oscillatory activity seems more informative here than evoked data (Tallon-Baudry and Bertrand 1999; Hagoort et al. 2004) and might reflect the sustained state and less time-locked neural responses of IFG compared with temporal regions (Liljeström et al. 2009). We note that BA44 and BA47 are distinct areas not only in their cytoarchitectonic organization (Amunts et al. 2010), but also in their pattern of long-range connectivity (Hickok and Poeppel 2007; Saur et al. 2010; Friederici 2012). A superior (dorsal) pathway along the arcuate fasciculus and the superior longitudinal fasciculus can be traced from the posterior part of the inferio-frontal gyrus (BA44 and BA45) to the parietal lobe (SMG) and toward temporal regions (pSTG and HG). In contrast, connecting tracts in the ventral stream are the extreme capsule (EmC) and the inferior longitudinal fasciculus, which connect the anterior part of the IFG (BA47 and to some extent BA45) with the anterior temporal gyri (aSTG and aMTG) and the posterior part of the perisylvian cortices (pSTG and pMTG; Saur et al. 2008). These dorsal and ventral structural streams have also been revealed at the functional level (Rolheiser et al. 2011; Griffiths et al. 2013). The dorsal pathway has been implicated in sensory-motor mapping processes (Hickok and Poeppel 2007), more precisely in analyzing sequences of segments and integrating them in a context (Rauschecker and Scott 2009) or even in integrating nonadjacent elements into syntactically complex structures (Friederici 2011). The ventral pathway has been taken to support sound to meaning mapping and is generally linked with semantic processing (Hickok and Poeppel 2007; Rolheiser et al. 2011; Friederici 2012) or with supporting combinations of adjacent elements in a sentence (Friederici 2012). The L-HG–L-BA47 synchrony observed in the current experiment can be related, both functionally and structurally, to the ventral pathway. The L-pSTG–L-BA44 synchrony, in contrast, is more likely to follow the dorsal pathway.

### Lexical Competition Within the Ventral Route

The gamma oscillation observed between L-HG and L-BA47 is common to both types of complex words, and consistent with the behavioral gating results. At early time points (−150 to −100 ms pre-onset closure), listeners are identifying the stem, with the gating data showing similar levels of competition for both types of complex words (Fig. 1*c* and see Supplementary Fig. 1). The L-HG–L-BA47 synchrony is also simultaneous with 2 other HG synchronies involving regions along ventral route (pSTS and pMTG), both previously related to mapping phonological information onto lexical representations (Hickok and Poeppel 2007; Friederici 2011). It is worth noting that the frontotemporal synchrony (L-HG–L-BA47) preceded the temporo-temporal ones, suggesting a role of frontal areas in integrating information due to prior knowledge (Sohoglu et al. 2012). Those results are consistent with involvement of BA47 in semantic processing (Mainy et al. 2008) and in lexical competition (Thompson-Schill et al. 1997; Bozic et al. 2010; Zhuang et al. 2014), and suggest a role of gamma oscillation in top-down processes (Tiitinen et al. 1993; Bertrand and Tallon-Baudry 2000), but see Arnal et al. (2011).

The second synchrony common to both types of complex words is the long-distance phase-locking between L-SMG and R-BA47. This interhemispheric synchrony could rely on the commissural fibers of the corpus callosum and the EmC on the right side of the brain (Saur et al. 2010), and may also involve ipsilateral and contralateral fibers from the auditory thalamic nuclei reaching the auditory cortices (Bartlett 2013). Since this synchrony occurs after the lexical competition is fully resolved (Fig. 1*c*), it may have only a secondary role compared with the L-HG–L-BA47 synchrony. However, the L-SMG cortices showed increased evoked activity during this synchrony (Fig. 3*a*) related to the processing of inflected words. L-SMG has been associated with phonological and not acoustic changes (Phillips 2001), implying that this brain area has access to already-abstracted phonological units and plays an important role in phonological working memory (Obleser and Eisner 2009). This suggests that phonological short-term memory is loaded more heavily for inflected words containing a grammatical morpheme that needs to be separated from its stem, and that performs different functions in the syntactic interpretation of an utterance.

### Morpho-phonological Parsing Within the Dorsal Route

The interaction between L-pSTG and L-BA44 is specific to the inflected words. This synchrony corresponds to the dorsal pathway, which has been related to phonological processing and mapping sound into articulatory representation (Hickok and Poeppel 2007; Friederici 2011; Rolheiser et al. 2011). The presence of an inflected form in English is accompanied by a specific phonological feature—an agreement in voicing between a final coronal consonant and the preceding segment (Marslen-Wilson and Tyler 2007). This co-occurrence effect could therefore be used to predict the presence of a syllable- or word-final grammatical morpheme, consistent with higher-level linguistic constraints. This gamma synchrony occurs as the preceding segment is being heard (before the onset closure) and suggests that rather than being a consequence of the suffix perception, the L-pSTG–L-BA44 long-range oscillation determines the linguistic interpretation of the word. Subsequent confirmation of the presence of the grammatical morpheme is accompanied by an increase of evoked activity within BA44 (Fig. 3*a*). Previous research has also linked local gamma modulation in the left BA44 with phonological processing (Mainy et al. 2008; Wheat et al. 2010). Our results suggest that this area has a role during phonological decoding that could precede modulation in the temporal pole (Wheat et al. 2010). Taken together, the posterior portion of the left IFG, BA44, may play a role in mediating between long-term phonological representations of motor articulation and short-term phonological representation in the sensory area (pSTG incorporating PT) (Rauschecker and Scott 2009). In parallel with phonological processing, left BA44 has been related to selection processes (Thompson-Schill et al. 1997; Zhuang et al. 2014). Zhuang et al. (2014) suggested a division of labor within the left IFG: Cohort size (the number of competitors sharing phonemes from word onset that are activated in parallel) increased neural activity in bilateral BA47, whereas cohort selection (reduction of competitors due to accumulation of acoustic information) focuses in left BA44. During the L-pSTG–L-BA44 synchrony, the stem of the inflected words has been fully heard and selected (Fig. 1*c* and see also Supplementary Fig. 1). This suggests a multifaceted role of left BA44 to inform and relay the information of inflected words to the sensory areas.

By employing noninvasive combined EMEG whole-brain recordings and a functional oscillation approach, our study provides a new perspective on how the human brain computes complex words. Our data provide support for models that emphasize morpho-phonological decomposition for linguistically complex words within the left perisylvian network, giving a central role to the left BA44. More generally, they highlight the intricate interaction between left frontal and temporal cortical areas through frequency-specific connectivity that underlies the neural computation of speech interpretation.

## Authors' Contributions

E.F., W.M.-W., and M.B. designed the research. E.F. collected the data, performed the analysis, and interpreted the EMEG data. E.F. wrote the manuscript with W.M.-W. and M.B.

## Supplementary Material

Supplementary material can be found at: http://www.cercor.oxfordjournals.org/.

## Funding

This work was supported by an EPSRC grant to W.M.-W. (EP/F030061/1), an ERC Advanced Grant (Neurolex) to W.M.-W., and by MRC Cognition and Brain Sciences Unit (CBU) funding to W.M.-W. (U.1055.04.002.00001.01). Computing resources were provided by the MRC-CBU. Funding to pay the Open Access publication charges for this article was provided by the Advanced Investigator Grant (Neurolex) to W.D.M.-W.

## Supplementary Material

Supplementary Data
