# Supplementary material for: Brain Network Connectivity During Language Comprehension: Interacting Linguistic and Perceptual Subsystems
Source: Cereb Cortex. 2014 Dec 1;25(10):3962–76. doi: 10.1093/cercor/bhu283 (PMC4585526; doi:10.1093/cercor/bhu283)
Supplement: Supplementary Data [file supp_25_10_3962__index.html]

Brain Network Connectivity During Language Comprehension: Interacting Linguistic and Perceptual Subsystems — Supplementary Data 

# Brain Network Connectivity During Language Comprehension: Interacting Linguistic and Perceptual Subsystems

## Supplementary Data

Supplementary Data

**Files in this Data Supplement:**

- Supplementary Data - Docx file
