# Supplementary material for: Brain Network Connectivity During Language Comprehension: Interacting Linguistic and Perceptual Subsystems
Source: Cereb Cortex. 2014 Dec 1;25(10):3962–76. doi: 10.1093/cercor/bhu283 (PMC4585526; doi:10.1093/cercor/bhu283)
Supplement: Supplementary Data [file supp_bhu283_bhu283supp.docx]

**Supplementary Material**

**Figure S1: Competition Ratio from the gating experiment**

A) Competition ratio by gate (see **Materials and Methods**) for the 3 types of words. Results showed a modulation of the level of competition depending on word type and gate, with a significant word type x gate interaction. B) Unpacking the interaction. Between -150 to 100 ms listeners are choosing the wrong stem for the perceptually complex words (*clay* instead of *claim*); however they are not yet aware that this guess is inaccurate. Between -75 to 0 ms pre onset closure, the level of competition for the embedded stem (*clay*) increases due to the accumulation of acoustic information that *clay* is not the target but *claim*. Inflected words at that stage have their stem (*play*) fully selected and recognised. After the alignment point (+25 and 100 ms), *clay* stem is not the preferred answer anymore and has a higher competition level compared to the inflected and simple words. Significant post-hoc analyses are coded with ***, *p*<.001; **, *p*<.01; *, *p*<.05.


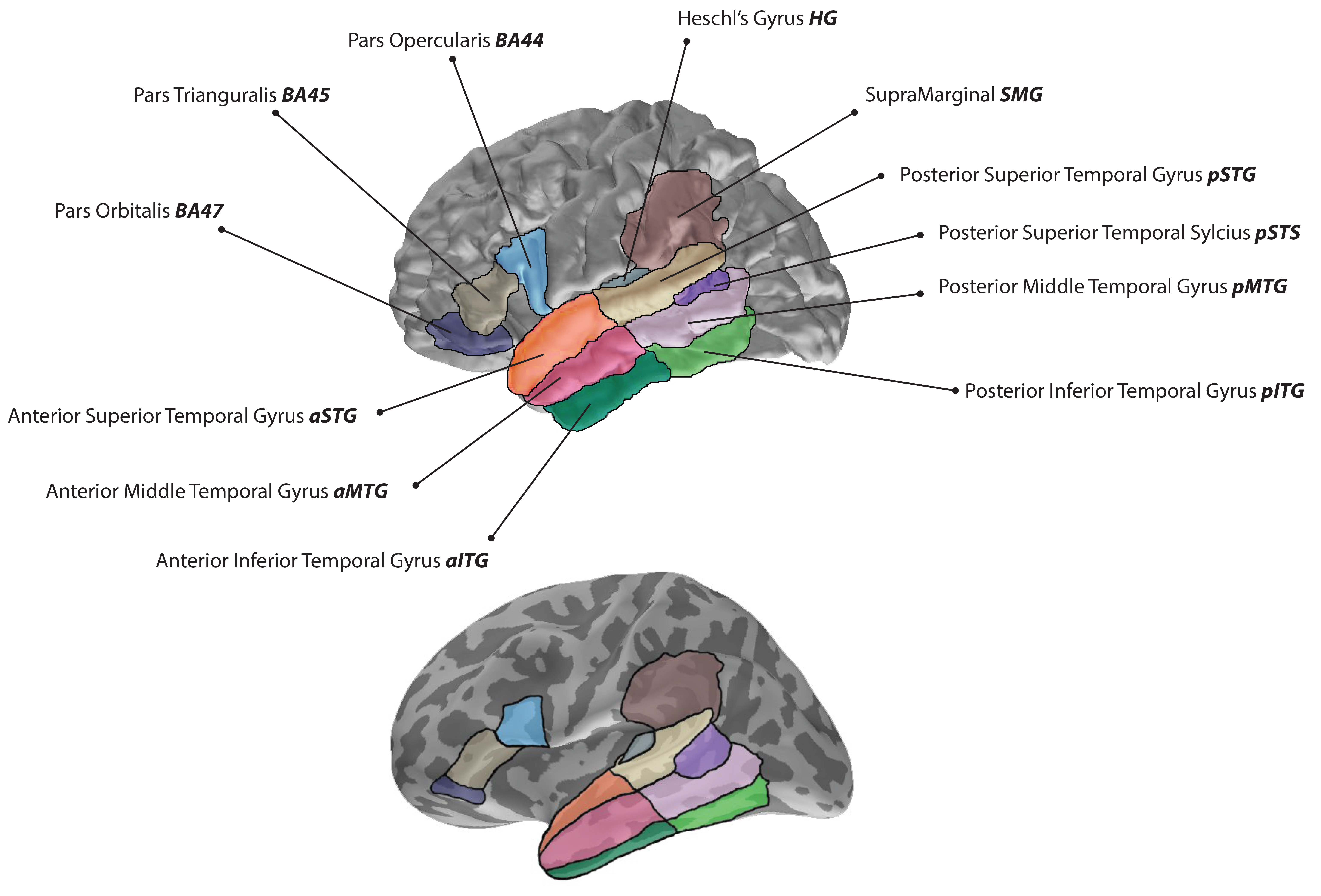


**Figure S2: Regions of Interest**

The 12 regions of interest per hemisphere used in the analysis, presented on the averaged cortical surface inflated (bottom) or not (top). They incorporated bilateral frontal (BA44, BA45, BA47) and temporal areas (Heschl’s gyrus, posterior superior temporal sylcus, supramarginal, superior, middle and inferior temporal gyri). Temporal gyri were divided into anterior and posterior ROIs.

**Figure S3: Waveform at onset closure and word onset for the 3 sensors**

Grand average waveforms (N=17), displayed at 6 representative locations for each sensor a) magnetometers, b) gradiometers, c) EEG. Negativity is plotted upwards. The distributions on the left display the data from onset closure alignment while the distributions on the right display the data from word onset alignment.

**Figure S4: Whole Brain MNE source estimate from onset closure**

a) Group average (N=17) source estimates (MNE) for the 3 types of words, averaged over the period of analysis around the onset closure (-200 to + 200 ms) and projected on the average inflated cortical surface (lateral view). b) Group average (N=17) source estimates (MNE) for the 3 types of words averaged over 50 ms time windows from -200 to +200 ms and for the left and right hemispheres. Results showed progressively increased amplitude of the neural activity in temporal and frontal areas over both hemispheres for simple and perceptually complex words (*shape* and *claim*) and left lateralised for linguistically complex words (*played).*

**Figure S5: Patterns of phase synchrony for perceptually complex words in the gamma range (20-60 Hz)**

A) Phase locking analysis showing significant trial by trial phase covariance between ROIs for the perceptually complex words (*claim*) compared to simple words (*shape*). All the synchronies involved the gamma band between 20 to 60 Hz. Colours code the start of the synchronies: purple between -200 to 150 ms, green between -150 to -100 ms, and orange after -50 ms. B) Details of the significant synchronies. Each significant cluster is represented by a box whose length corresponds to its timing. *P* values (*p*<.05, corrected for multiple comparisons) and frequency band (min-max/peak in Hz) are indicated inside the box. The average latency of maximum difference between the perceptually complex word (*claim*) and simple word (*shape*) is reported (mean: black circle; ± s.e.m horizontal bar).


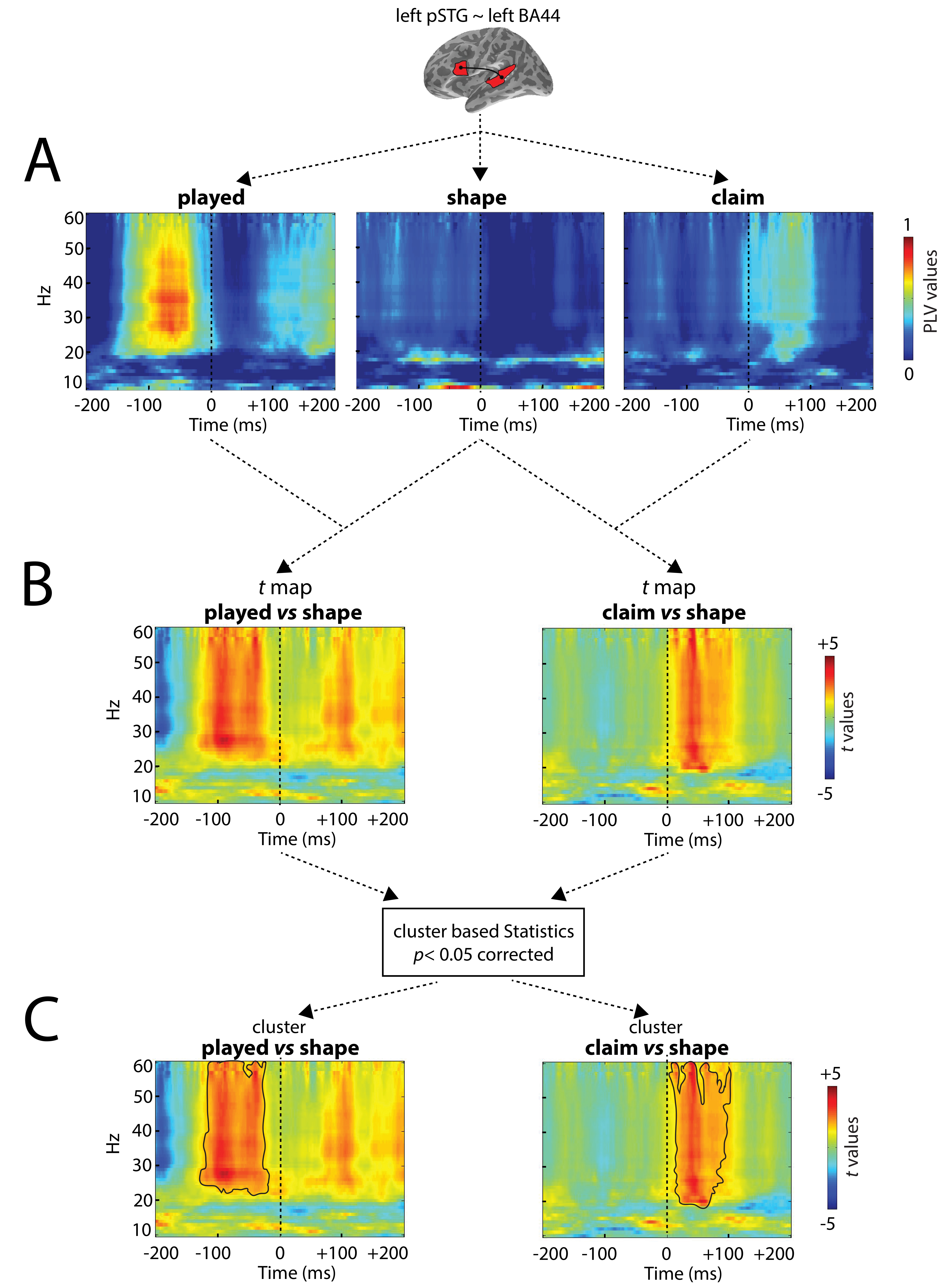


**Figure S6: Phase locking analysis through all frequencies**

Overview of the processing stream used to compute the phase-locking analysis. All the charts are grand average results across subjects. A) The phase-locking values between two ROIs are computed for a specific word type. PLVs range from 0 (random) to 1 (aligned) and are inversely correlated with the variance in the trial-by-trial phase difference between the 2 signals. B) Difference of PLV between conditions is statistically determined using paired-samples *t* tests for each frequency and time point. C) Cluster-mass permutation tests (10 000 permutations) with an alpha level of .05 (one-tailed) were used to determine the significance of each cluster and to control for multiple comparisons.

**Table S1: List of the experimental stimuli**

The study used 240 words divided into 3 test conditions (80 words each): regular past tense (*played*), words with onset-embedded stem (*claim*) and simple words (*shape*).

***N* x y t/ms time window/ms  *Z* *p***

**magnetometers**

18648 0 21 -46 -130 +30 5.49 0.0000004

10032 -21 -12 -62 -110 +115 6.12 0.0000004

**gradiometers**

3329 -30 6 -2 -30 +35 4.72 0.00001

2256 33 -9 170 +145 +200 4.89 0.000001

**EEG**

28949 -6 3 -108 -145 +45 6.43 0.0000003

18989 -42 -18 -96 -135 +105 6.45 0.0000003

**Table S2: Description of the SPM ‘space x time’ results for the omnibus ANOVA effects, RFT-corrected *p*<.001**

The origin of SPM coordinates is the midpoint of a square image, with x ranging from −48 (left) to +48 (right) and y ranging from −39 (posterior) to +45 (anterior). t refers to peristimulus time; Z=Z-score, N=number of “voxels”. Note that only the global maximum is shown for main effect of condition (other clusters showing a condition effect are available on request).

amplitude T-test

___________________________ __________________________________

played claim shape played claim

*vs.* *vs.*

claim shape

_________________________________________________________________________________________

*Played clusters*

**Left**

1. L-HG 0.25 *(.02)* 0.19 *(.01)* 0.17 *(.01)* .01
2. L-SMG 0.19 *(.01)* 0.17 *(.009)* 0.15 *(.009)* .03
3. L-pSTG 0.26 *(.01)* 0.21 *(.01)* 0.19 *(.01)* .002

4a) L-pMTG (*cl1*) 0.21 *(.01)* 0.19 *(.01)* 0.17 *(.01)*

4b) L-pMTG (*cl2*) 0.25 *(.01)* 0.21 *(.01)* 0.20 *(.01)* .02

1. L-aSTG 0.28 *(.01)* 0.25 *(.01)* 0.22 *(.01)*
2. L-aMTG 0.40 *(.02)* 0.35 *(.01)* 0.32 *(.02)* .01
3. L-BA44 0.28 *(.02)* 0.24 *(.01)* 0.23 *(.01)* .04

**Right**

1. R-HG 0.20 *(.02)* 0.16 *(.01)* 0.16 *(.02)*

**Table S3: Source activity related to the processing of an inflected word**

Mean amplitude of signal change (*SE*) for the 3 types of words for each region of interest (8) showing a significant cluster for the comparison *played* vs. *shape*. Those values have been entered into a *t*-test for dependent samples (*played vs.* *claim* and *claim* vs. *shape*). We report only significant *p* values (*p* <.05).

% PLV change T-test

______________________ ______________________________

played claim shape played claim

*vs.* *vs.*

claim shape

________________________________________________________________________________________

*Played clusters*

1. L-HG ~ L-BA47 17.49 13.20 - 24.15 .01
2. L-HG ~ L-pSTS 32.33 6.21 -11.16
3. L-pSTG ~ R-aMTG 23.65 4.62 -15.60
4. L-pSTG ~ L-BA44 55.09 11.10 14.31 .01
5. L-aMTG ~ L-BA45 30.13 35.67 -6.32
6. L-BA44 ~ R-SMG 38.91 7.75 0.24
7. L-HG ~ L-pMTG 41.05 17.22 2.88
8. L-SMG ~ R-BA47 35.23 34.79 -8.75 .04
9. L-aSTG ~ R-aSTG 24.88 9.21 -16.94
10. L-HG ~ R-HG 27.01 6.50 -9.77

**Table S4: Mean percentage of gamma phase-locking change related to the baseline**

Mean percentage of gamma phase-locking change related to the baseline (-300 -200 ms) for the 3 types of words and for the 10 significant clusters obtained in the analysis comparing *played* vs. *shape*. These values have been entered into a *t*-test for dependent samples (*played vs.* *claim* and *claim* vs. *shape*). We report only significant p values (*p* <.05).
